# Anti-Inflammatory Effects of Arsenic Trioxide Eluting Stents in a Porcine Coronary Model

**DOI:** 10.1155/2013/937936

**Published:** 2013-01-16

**Authors:** Li Shen, Feirong Gong, Wenjie Tian, Weiming Li, Feng Zhang, Juying Qian, Aijun Sun, Yunzeng Zou, Wei Yang, Junbo Ge

**Affiliations:** ^1^Shanghai Institute of Cardiovascular Diseases, Zhongshan Hospital, Fudan University, Shanghai 200032, China; ^2^Key Laboratory for Ultrafine Materials of Ministry of Education, School of Materials Science and Engineering, East China University of Science and Technology, Shanghai 200237, China; ^3^Department of Cardiology, Sichuan Provincial People's Hospital, Chengdu 610072, China; ^4^Department of Cardiology, The First Affiliated Hospital of Harbin Medical University, Harbin, Heilongjiang 150001, China

## Abstract

Previous research from our group has demonstrated arsenic trioxide eluting stents significantly reduced neointimal area and thickness compared with bare metal stents. In the present study, the anti-inflammatory effects of arsenic trioxide *in vitro* and arsenic trioxide eluting stents in a porcine coronary model have been explored. Sixty-five pigs underwent placement of 139 oversized stents in the coronary arteries with histologic analysis, endothelial function analysis, and immunohistochemical and western blot analyses. Arsenic trioxide eluting stents effectively inhibited local inflammatory reactions, while no significant difference in endothelialization and endothelial function between arsenic trioxide eluting stents and bare metal stents was observed. Arsenic trioxide eluting stents favorably modulate neointimal formation due to less augmentation of early inflammatory reactions, and quick endothelialization of the stent surface, which might contribute to long-term safety and efficacy of drug eluting stents.

## 1. Introduction

Polymer-based sirolimus and paclitaxel drug eluting stents (DESs) have revolutionized the treatment of coronary artery diseases through remarkable reduction of angiographic target lesion revascularization [1, 2]. In real world clinical settings, however, concerns about long-term safety and efficacy have emerged [3–6]. The first-generation sirolimus and paclitaxel eluting stents are both associated with incomplete neointimal coverage [7, 8], impaired endothelial cell function [9], thrombosis [10], hypersensitivity reactions [11], and incomplete stent apposition [12]. Currently used drugs (sirolimus and paclitaxel) and nondegradable polymer coatings are mostly considered to be responsible for late adverse events and pathologic reactions, including the late stent thrombosis, inflammation, and hypersensitivity [13, 14]. 

DESs are designed to reduce in-stent neointimal growth through the elution of agents that arrest the cycle of cell proliferation. An ideal DES drug should suppress excessive neointimal growth while maintaining the proliferation of endothelial cells and functioning endothelium [15]. Arsenic trioxide (As_2_O_3_) has been used in the treatment of patients with acute promyelocytic leukemia (APL) [16, 17] and other types of malignant cancers [18–22]. As_2_O_3_ acts on cells through a variety of mechanisms, influencing numerous signal transduction pathways and resulting in a vast range of cellular effects that include apoptosis induction, growth inhibition, promotion or inhibition of differentiation, and angiogenesis inhibition [23, 24]. In a recent report [25], a heparin-immobilized copolymer of L-lactide (LA) and 5-methyl-5-benzyloxycarbonate-1,3-dioxan-2-one (MBC) has been synthesized as a biodegradable coating material for local As_2_O_3_ delivery. This copolymer was found to reduce thrombosis, localized hypersensitivity, and inflammation, which belong to late-stage adverse events.

The pathological process of in-stent restenosis (ISR) is characterized by an inflammatory healing response after stretch and damage of the vessel wall [26–34]. Stent restenotic reduction of DESs can be mainly ascribed to its antiproliferative activity, but anti-inflammation may also be an important explanation for controlling inflammation-triggered proliferative process [35, 36]. As_2_O_3_ eluting stents (AESs) effectively reduce neointimal thickening in rabbit and porcine models through inhibiting the cell cycle and inducing vascular smooth muscle cell (VSMC) apoptosis [25, 37]. The present study was performed to evaluate the behavior of inflammatory cells to As_2_O_3_ and effect of AESs in a porcine coronary model, thus reveal the mechanism of AESs in reducing ISR. 

## 2. Materials and Methods

### 2.1. Animals

Pigs (~20 kg weight) were obtained from the Shanghai Animal Administration Center and received daily oral antiplatelet medication until termination. All animal experiments were approved by the Animal Care and Use Committee of Fudan University and were in compliance with the “Guide for the Care and Use of Laboratory Animals” published by the National Academy Press (NIH Publication No. 85-23, revised in 1996). 

### 2.2. Preparation and Surface Morphology of AESs

Heparinized polymer solution [37] (0.1 wt.% in dichloromethane), As_2_O_3_ aqueous solution (1 wt.%), and heparinized polymer solution (0.1 wt.% in dichloromethane) were sequentially sprayed onto the surface of BMSs (3.0 × 17 mm, Beijing Amsinomed Medical Company, China) to form the basecoat (20 ± 3 *μ*g), drug layer (40 ± 5 *μ*g), and topcoat (40 ± 5 *μ*g), respectively. Polymer-coated stents (PCSs) were prepared by directly spraying the polymer solution onto the stent surface and the quantity of polymer was 60 ± 5 *μ*g per stent. 

### 2.3. Stent Implantation

On the procedure day, thirty male pigs were anesthetized with ketamine (20 mg/kg intramuscularly) and xylazine (2 mg/kg intramuscularly). BMSs (*n* = 21, 7 for one week, 5 for two weeks, and 9 for four weeks), PCSs (*n* = 16, 5 for one week, 5 for two weeks, and 6 for four weeks), and AESs (*n* = 32, 9 for one week, 11 for two weeks, and 12 for four weeks) were implanted in 2-3 coronary arteries per pig by random assignment to anatomic location. The resulting stent-to-artery ratio was about 1.2-1.3 : 1 by quantitative coronary angiography analysis. The animals were anesthetized with ketamine (20 mg/kg) and xylazine (2 mg/kg) for follow-up angiography in the same orthogonal views before death with 20 mL of potassium chloride intracoronary injection. The stented arteries were carefully dissected from the myocardium and cut into two pieces, each about 9 mm long for cross sections preparation and SEM imaging. 

### 2.4. Measurements of As_2_O_3_ Levels in Tissues and Serum

In additional animals at 0, 4, 24, 72 h, and 7 days after deployment of As_2_O_3_ eluting stents (*n* = 6, 6 stents per time point) in the coronary arteries, the delivery of As_2_O_3_ from stents to tissues and serum was evaluated using hydride generation reaction interfaced with atomic fluorescence spectrometry assay and HPLC, respectively [37]. In this experiment, fifteen pigs were used and each pig received two As_2_O_3_ eluting stents. 

### 2.5. Endothelialization of Stented Arteries and Evaluation of Endothelial Function

The endothelialization of stented arteries was examined using SEM 1, 2, and 4 weeks after stent implantation. Endothelial function after stent implantation was estimated by measuring the coronary vasomotor reactivity in response to acetylcholine (Ach, 60 mg, performed at an infusion rate of 1 mL/min) infusion within 6-month followup. Ten pigs receiving two stents each and 20 stents (6 BMSs, 7 PCSs, and 7 AESs) were used in the experiment. End-diastolic images for each segment were chosen and analyzed with the automated edge detection program (FD-10, Philips, Best, The Netherland). Two orthogonal views with less foreshortening or without overlapping of side branches were selected and averaged for biplane assessment by two experts blinded to stent type. About 5 mm distal to the site of stenting was chosen for analysis. Changes in coronary diameter in response to Ach coronary infusion were expressed as percent changes versus baseline angiograms.

### 2.6. Immunohistochemical and Western Blot Analyses

For immunohistochemical and western blot analyses, ten pigs were used and each pig randomized received two stents (7 BMSs, 6 PCSs, and 7 AESs). After 4 weeks, the stented arteries were harvested and cut into two pieces. The stent struts were carefully removed from the coronary arteries. Serial sections from the stented arteries were fixed in phosphate buffered saline (PBS) containing 10% formalin, dehydrated by treatment with solutions of increasing alcohol content (70%, 85%, 95%, and 100%), followed by xylene and then embedded in paraffin. Immunohistochemical studies were performed using primary antibodies against interleukin-6 (IL-6), monocyte chemoattractant protein-1 (MCP-1), CD3, and S100. Briefly, the sections were deparaffinized, heated at 95°C for 30 min in antigen retrieval solution, fixed in 0.3% hydrogen peroxide for 30 min, and then air dried. Nonspecific antibody binding sites were blocked by a 30 min incubation in normal horse serum (10%, 0.1% Triton X-100, 0.1 M PBS, pH 7.4). The slide was then incubated in the primary antibodies, respectively. (1 : 100, 0.1% Triton X-100, 0.1 M PBS) (R&D systems, Minneapolis, MN, USA) overnight at 4°C. After three washes in PBS, the slides were again blocked in normal horse serum as described above and exposed to the horseradish peroxidase-conjugated secondary antibody (1 : 400, 0.1% Triton X-100, 0.1 M PBS) (Beyotime, China), followed by the addition of 3,30-diaminobenzidine tetrahydrochloride (DAB) solution. Nuclei were also counterstained with hematoxylin. The total tissue area of the immunostained sections of each specimen was outlined manually and measured in mm^2^. The tissue areas of the immunostained sections occupied by positive cells were measured automatically using greyscale detection with a fixed threshold and the ratios of immunopositive areas were calculated as percentages of the total tissue area.

Vessel wall expression of inflammatory-associated proteins (IL-6 and MCP-1) was determined by western blot analysis. Briefly, protein extracts (50 *μ*g) were size fractionated on SDS-polyacrylamide gels, transferred to nitrocellulose membrane. Positive control for each target was run on the same gel. Membranes were incubated with an affinity purified polyclonal antibody to IL-6 and MCP-1, respectively, washed, and incubated with secondary antibody. Signals were detected by the ECL chemiluminescence detection system. Glyceraldehyde-3-phosphate dehydrogenase (GAPDH) was used as an internal control to ensure equal amount of protein extract in each sample. 

### 2.7. Statistical Analysis

Numerical data are presented as mean ± standard error of the mean. Continuous variables were compared by ANOVA (*t*-test with Bonferroni correction), and categorical variables were compared by *χ* ^2^ test. A *P* value of ≤ 0.05 was considered as a significant difference.

## 3. Results

### 3.1. Surface Morphology Examination of AESs

**Figure 1:**
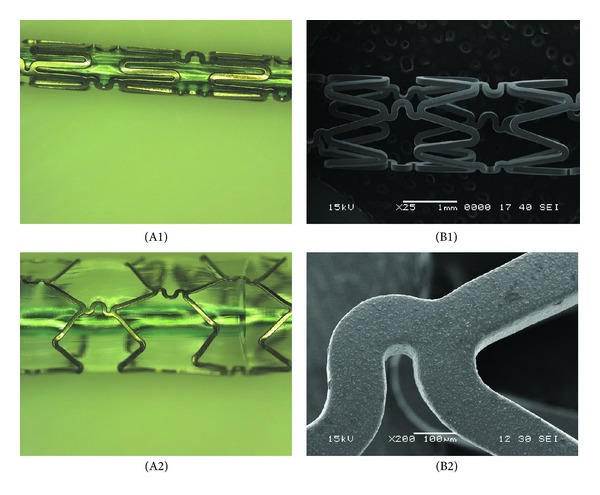
Digital photographs (A) and SEM images (B) of As_2_O_3_ eluting stent before (1) and after (2) expansion.

Figure 1 shows the stereoscopic and SEM images of an As_2_O_3_ eluting stent before and after expansion. It can be clearly observed that As_2_O_3_ crystals were dispersed between the basecoat and topcoat, while the coatings were still uniform. No delamination or destruction of the coatings on the stent can be observed, indicating the drug does not impair integrity of the coatings. 

### 3.2. *In Vivo* As_2_O_3_ Levels after Stent Implantation

The levels of As_2_O_3_ measured in arterial tissue, serum, and main organs after AESs implantation are presented in Table 1. At 4 h after stent deployment, the mean concentration of As_2_O_3_ within the adjacent arterial tissue was 1.264 ± 0.056 *μ*g/g, whereas the value in serum was 0.012 ± 0.004 *μ*g/g, nearly one hundred times higher than that in the serum. The concentration of As_2_O_3_ in stented artery tissue reached a maximum level at 24 h, and decreased thereafter, whereas the values in serum, heart, liver, and kidney progressively declined after 4 h. Even at day 7 after stent implantation, the As_2_O_3_ concentration in the arterial wall was 0.036 ± 0.002 *μ*g/g, while no As_2_O_3_ can be detected in the serum, heart, liver, and kidney. 

### 3.3. Morphological Evaluation of the Stented Arteries

**Figure 2:**
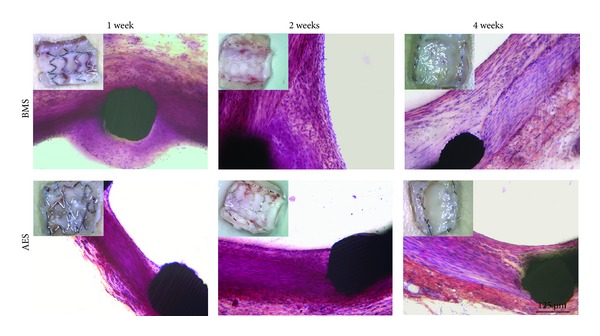
Typical high-power micrographs 1 week, 2 and 4 weeks after the placement of BMSs and AESs in normal porcine coronary arteries. Insert: Digital camera photographs of the inner wall of the stented arteries. At the first week, a thick layer of matrix consists of a lot of inflammatory cells and fibrin associated with foreign body reactions is present over the stent struts for BMSs. A much thinner layer of such matrix can be observed for AESs. At 2 weeks, the neointima consisting of amorphous material and inflammatory cells formed. This amorphous material adjacent to the stent strut was never observed 2 weeks after AESs implantation. At 4 weeks, the neointima consists of smooth muscle cells and matrix proteoglycans. The density of smooth muscle cells in neointima is much lower for AESs than BMSs.

The typical morphologies of the stented arteries 1, 2, and 4 weeks after stent implantation are shown in Figure 2. In the first two weeks, BMSs and AESs had different appearance with fibrin-platelet deposition and acute inflammatory cells. At one week, a relatively thick layer of matrix composed of many inflammatory cells and fibrin-associated foreign body reactions was present over the stent struts for BMSs, while a much thinner layer of such matrix was observed in the AES group. The percent area stenosis for BMSs and AESs at this time point was 7.6 ± 2.7% and 4.3 ± 1.6%, respectively. After two weeks, neointima consisting of amorphous material and inflammatory cells formed. The amorphous material adjacent to the stent strut was also less for AESs as compared with the controlled BMSs. After 4 weeks, neointima consists of SMCs and matrix proteoglycans formed. The density of SMCs in neointima was much lower in the AES group than in the BMS group. There was a trend that the percent area stenosis at four weeks was lower in the AES group compared to BMS, although the difference was not significant (*P* = 0.075). 

### 3.4. Endothelialization of the Stented Arteries and Evaluation of Endothelial Function

**Figure 3:**
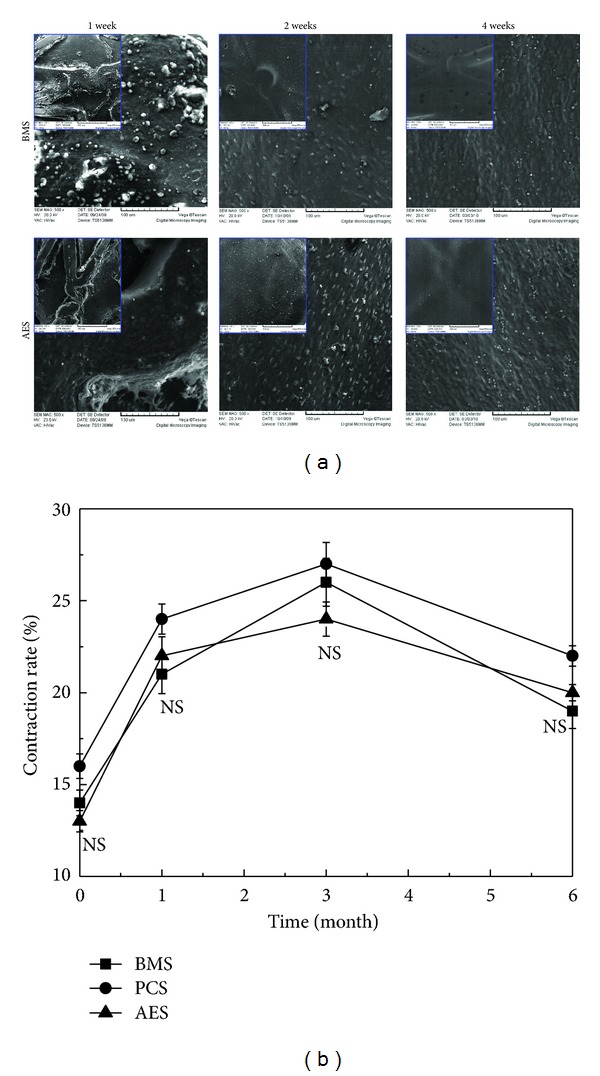
Typical SEM images (magnification: ×500, ×100 for insets) of the inner wall of stented arteries 1, 2, and 4 weeks after implantation (a) and relaxation responses of coronary segments distal to stents within 6-month followup (b). At the first week, both AESs and BMSs showed quite few endothelial cells attachment. For the second week of development, the endothelialization of AESs was slightly higher than BMSs (*P* > 0.05). After 4 weeks, the arteries treated with BMS or AESs were fully endothelized. The diameter changes between the BMSs, PCSs, and AESs had no significant differences of vasoreactivity in response to the Ach infusion in sites distal to the stents within 6-month followup.

The progress of endothelialization of stented arteries was examined using SEM at 1, 2, and 4 weeks after stent implantation as shown in Figure 3(a). At the first week, both AES and BMS had quite low endothelial cells attachment. AES showed much less fibrin-platelet deposition than BMS, which was consistent with the morphological results in Figure 2. For the second week of development, endothelial cell attachment could be found to occur on the BMS surface with a relative low density. Although AESs were reported to mildly delay endothelialization in a rabbit iliac artery injury model [25], no difference was observed in a porcine coronary model. After 4 weeks, there was no difference in endothelialization for AES and BMS. The arteries treated with BMSs or AESs were fully endothelialized; the lumen surface of the vessel wall and the stent struts were covered by confluent endothelial cells. 

In this study, the diameter changes in response to the Ach infusion in sites distal to the stents between BMSs, PCSs, and AESs had no significant differences within 6-month followup (Figure 3(b)). 

### 3.5. Immunohistochemical and Western Blotting Analyses

**Figure 4:**
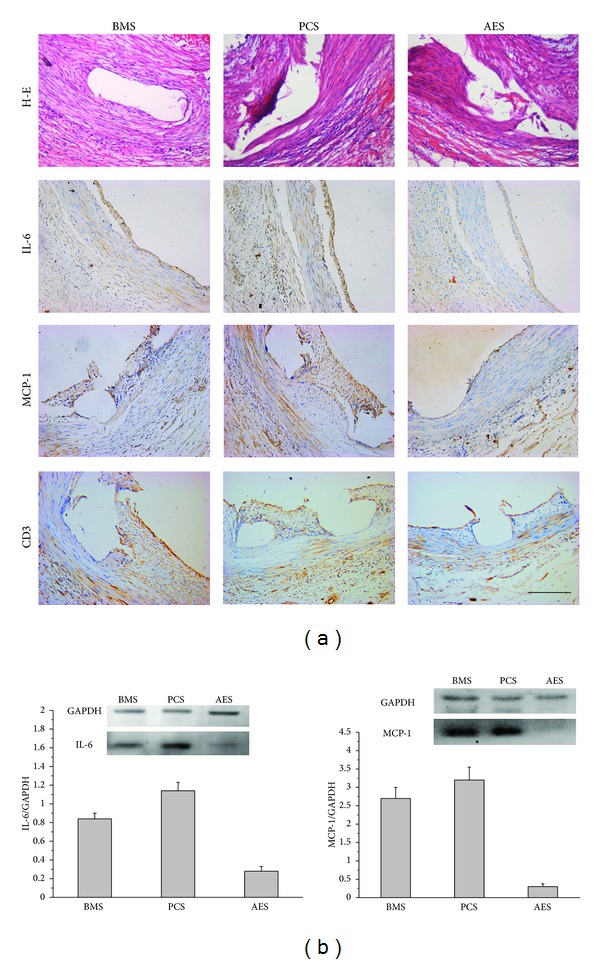
Immunohistochemical staining (a) and western blotting analysis (b) of arteries treated with BMSs, PCSs, and AESs for 4 weeks after stent implantation. The stent-based As_2_O_3_ delivery significantly reduced the expression of proteins MCP-1, IL-6, and CD3 (*P* < 0.05). In western blotting analysis, the expression of MCP-1 and IL-6 in the arteries treated with AESs was significantly lower than BMSs and PCSs (*P* < 0.05).

Figure 4 shows the immunohistochemical staining and western blotting results 4 weeks after stent implantation. Stent-based As_2_O_3_ delivery significantly (*P* < 0.05) inhibited MCP-1, IL-6, and CD3 expression. The ratios of MCP-1, IL-6, and CD3 positive area for BMS, PCS, and AES were 15.4 ± 5.3, 17.4 ± 4.5, 8.2 ± 2.6%; 22.4 ± 6.6, 25.2 ± 7.3, 5.2 ± 3.6%; and 20.6 ± 4.1, 19.8 ± 4.5, 6.7 ± 2.9%, respectively, in Figure 4(a). Immunohistochemical analysis failed to detect any difference in the expression of S100 between BMS, PCS, and AES 4 weeks after stent implantation. We further used antibodies for MCP-1 and IL-6 in western blotting analysis as demonstrated in Figure 4(b). Vascular segments treated with PCSs demonstrated a slight increase in MCP-1 and IL-6 compared with the BMSs control group (*P* > 0.05). The expression levels of MCP-1 and IL-6 in the arteries treated with AESs were significantly lower than those treated with BMS and PCS (*P* < 0.05). 

## 4. Discussion

It is well known that ISR is a consequence of inflammation, SMC proliferation, and migration [38]. The effects of As_2_O_3_ as an antiproliferative agent for drug eluting stents at clinically achievable concentrations were investigated without severe side effects. In the studies done in rabbit iliac artery model and porcine coronary model, AESs effectively reduce neointimal thickening through inhibiting the cell cycle and inducing VSMC apoptosis. No in-stent thrombosis can be observed within six-month followup in porcine coronary arteries [25, 37], suggesting that AESs are a feasible, safe, and efficient DES. In the present study, anti-inflammatory effect of As_2_O_3_ *in vitro* and effect of AESs in a porcine coronary model have been evaluated. 

AESs were also found to significantly decrease the protein expression of MCP-1, IL-6, and CD3 compared with BMSs and PCSs. But unfortunately, we failed to detect any decrease of S100 expression, a marker of DCs 4 weeks after AESs implantation. Indeed, the expression of S100 in BMS, PCS, and AES treated arteries was low and the difference was not significant at this time point. MCP-1 is the prototype of the C-C chemokine-*β* subfamily and exhibits its most potent chemotactic activity toward monocytes and T lymphocytes. In addition to promoting the transmigration of circulating monocytes into tissue, MCP-1 exerts various other effects on monocytes, including superoxide anion induction, cytokines production, and adhesion molecule expression [39]. IL-6 is a cytokine that has been proven to be crucially involved in ischemic heart disease. This substance possesses pleiotropic functions that are mostly in favor of inflammation. Patients with a higher release of IL-6 in the coronary sinus after stent deployment have a higher risk of restenosis. Suzuki and coworkers [40] showed in an experimental model that sirolimus- and dexamethasone-covered stents induce a reduction in release of MCP-1 and IL-6 compared with BMSs, thus decreasing local inflammation. 

Endothelium plays an integral role in maintaining vascular homeostasis. This role is not limited to the modulation of vascular tone but also for regulation of inflammation, platelet activation, and thrombosis [41, 42]. Drug eluting stents substantially reduce in-stent restenosis in patients with coronary artery disease and also lead to disruption of the endothelial layer and leaves a thrombogenic metallic surface exposed to the blood stream. Early DES devices have been associated with late stent thrombosis (LST) in patients, a rare though life-threatening event that has become an increasingly controversial issue for interventional cardiology. Delayed or incomplete re-endothelialization is an important predictor of LST, which is increasingly apparent for DES in real-world applications. The early establishment of a functional endothelial layer after vascular injury has been shown to assist in prevention of neointimal proliferation and thrombus formation [43, 44]. In our previous report, stent thrombosis did not occur within 6-month followup for the AESs in a porcine coronary artery model [37]. Stent-based local delivery of sirolimus (Cypher) and paclitaxel (Taxus) profoundly inhibited neointima formation but caused vasomotor dysfunction in distal conduit vessel segments [44]. As revealed in previous reports, the segments proximal and distal to the stents were both more strongly constricted to Ach infusion in the SES group compared with the BMS group [45–47]. There was no significant difference in extensive endothelialization and endothelial function between AESs and BMSs after 4 weeks, suggesting improved safety of AES than SES and PES. The early restoration of endothelial function after AES implantation might be interpreted as the result of rapid elution of the loaded drug, which was completely released within 7 days, and thus minimized local toxicity [37]. In contrast, the loaded drug for sirolimus and paclitaxel eluting stents is released up to 60 days after stent implantation. Another reasonable explanation is related to the biodegradable coating material that can induce mild foreign body reactions and quick endothelialization [37]. 

## 5. Conclusions

In a porcine coronary artery model AES reduced the neointimal hyperplastic response to injury through inhibition of cell cycle and induction of apoptosis of VSMC [27, 48], less augmentation of early inflammatory reactions, and quick endothelialization of the stent surface. Although further studies are necessary to confirm the long-term efficacy and safety of this novel medical device, our data highlight the necessity to improve our understanding regarding the performance of AESs in reducing ISR. 

## Figures and Tables

**Table 1:** As_2_O_3_ levels in serum and tissues.

	0 h (*μ*g/g)	4 h (*μ*g/g)	24 h (*μ*g/g)	72 h (*μ*g/g)	7 days (*μ*g/g)
Serum	<0.001	0.012 ± 0.004	0.010 ± 0.003	0.007 ± 0.001	<0.001
Heart	<0.001	0.048 ± 0.002	<0.001	<0.001	<0.001
Liver	<0.001	0.032 ± 0.014	0.027 ± 0.012	0.021 ± 0.004	<0.001
Kidney	<0.001	0.12 ± 0.027	0.094 ± 0.008	0.054 ± 0.0021	<0.001
Stented artery	<0.001	1.264 ± 0.056	1.767 ± 0.052	0.475 ± 0.007	0.036 ± 0.002
